# Recent Advances and Uses of Grape Flavonoids as Nutraceuticals

**DOI:** 10.3390/nu6010391

**Published:** 2014-01-21

**Authors:** Vasil Georgiev, Anthony Ananga, Violeta Tsolova

**Affiliations:** Center for Viticulture and Small Fruit Research, College of Agriculture and Food Sciences, Florida A & M University, 6505 Mahan Drive, Tallahassee, FL 32317, USA; E-Mails: anthony.ananga@gmail.com (A.A.); violetka.colova@famu.edu (V.T.)

**Keywords:** antioxidants, anti-inflammation, grapevine, polyphenols

## Abstract

Grape is one of the oldest fruit crops domesticated by humans. The numerous uses of grape in making wine, beverages, jelly, and other products, has made it one of the most economically important plants worldwide. The complex phytochemistry of the berry is characterized by a wide variety of compounds, most of which have been demonstrated to have therapeutic or health promoting properties. Among them, flavonoids are the most abundant and widely studied, and have enjoyed greater attention among grape researchers in the last century. Recent studies have shown that the beneficial health effects promoted by consumption of grape and grape products are attributed to the unique mix of polyphenolic compounds. As the largest group of grape polyphenols, flavonoids are the main candidates considered to have biological properties, including but not limited to antioxidant, anti-inflammatory, anti-cancer, antimicrobial, antiviral, cardioprotective, neuroprotective, and hepatoprotective activities. Here, we discuss the recent scientific advances supporting the beneficial health qualities of grape and grape-derived products, mechanisms of their biological activity, bioavailability, and their uses as nutraceuticals. The advantages of modern plant cell based biotechnology as an alternative method for production of grape nutraceuticals and improvement of their health qualities are also discussed.

## 1. Introduction

Grape (*Vitis* spp.) is one of the most economically important plant species due to its diverse uses in production of wine, grape juice and other food products [1]. It is cultivated in all continents in the temperate regions where sufficient rain, warm and dry summers as well as relatively mild winters are normal climatic patterns [2]. The qualities of grape products are characterized by their metabolic compositions. Flavonoids represent a widespread and common group of natural polyphenols produced by the phenylpropanoid pathway [3,4]. They confer UV-protection, determine flower coloration, attract pollinators, and act as tissue protectors in case of pathogen attack or oxidative damage [5]. In grapes, flavonoids are primarily located in the epidermal layer of berry skin and the seeds [6,7,8,9]. Flavonoids are the main groups of soluble phenolics in grapes as well as major contributors of the biological activities in products derived from grapes [10].

Recently, the strong beneficial health effect of grape flavonoids has been directly connected to the so called “French Paradox”. The term refers to the epidemiological observation of comparatively low incidence of coronary heart disease in the population of the Mediterranean region, despite the presence of a local diet rich in saturated fats. The tradition of regular consumption of red wine which lowers the risk for development of cardiovascular diseases was reported as the main contributing factor [11]. Flavonoids in red wine are the most feasible phytochemicals, responsible for this phenomenon [12]. Flavonoids have cardioprotective, antioxidant, anti-inflammatory, anti-cancer and antimicrobial properties, and are one of the most potent nutraceuticals in food and phytopharmaceutical products [13,14,15,16,17,18]. Therefore, it is paramount to understand the principles of biological activity, bioavailability and metabolism of grape flavonoids in relation to human health.

## 2. Biological Activities of Grape Products

**Figure 1:**
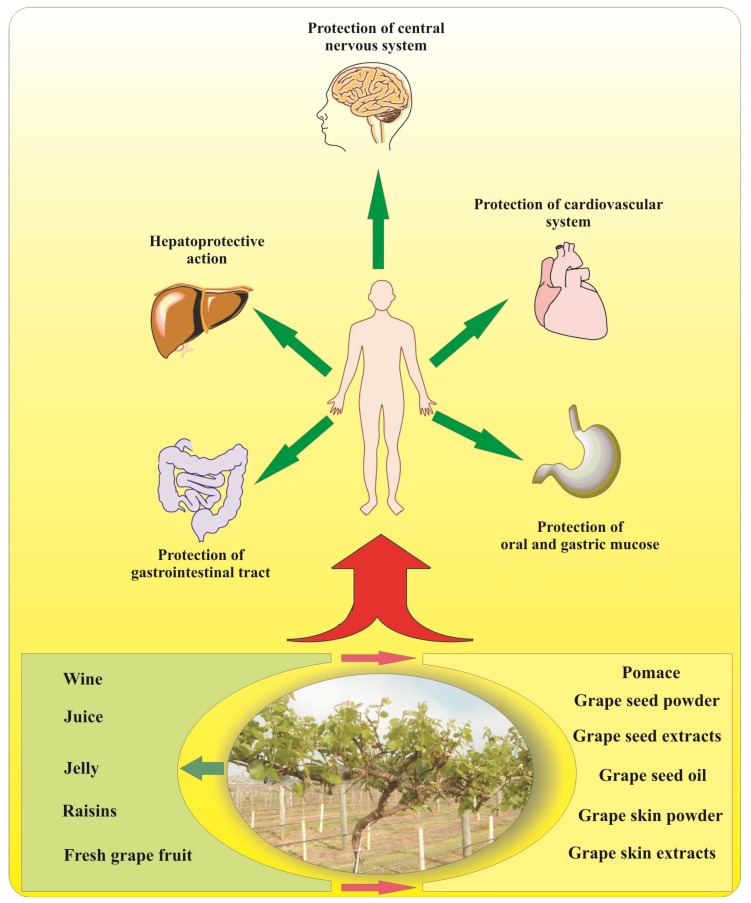
Primary grape products and byproducts and their beneficial effects on human body.

Wine, the product of grape juice fermentation has played an important role in the development of human culture. The earliest chemical evidence for wine production was found in the Middle East in well-preserved ancient jars dated 5400–5000 BC [19]. The multitude of ancient and historical images of vines and grapes found as decorative elements on ancient coins, temples, ritual potteries, and mosaic sculptures clearly demonstrated the importance of grape and its products in ancient societies across the globe. For example, in the Balkans and the Eastern Mediterranean, grape and wine were considered divine and dedicated to various deities: “Zagreus” by Thracians, “Dionysus” by Greeks and “Bacchus” by the Romans [20,21,22]. The mystical powers of grape and wine in the social life and cultural traditions of ancient people were not futile. Modern science continues to decipher the benefits of grape as a rich source of valuable phytonutrients with remarkable positive effects on human health [1,23,24,25,26,27]. The unique combination of phytochemicals in grapes includes a variety of bioactive compounds such as simple phenolics, flavonoids, anthocyanins, stilbenes, proanthocyanidins, and vitamin E [1,3,25]. In excess of 500 compounds, including 160 esters, have been identified to be present in wines with active role in the formation of their organoleptic properties [1]. Simple phenolics in grapes are derivatives of hydroxycinnamic acid (p-coumaric, caffeic, sinapic and ferulic acids) and hydroxybenzoic acid (gallic, gentisic, protocatechuic and p-hydroxybenzoic acids) [1]. In wine, hydroxycinnamic acid derivatives are found as esters with tartaric acid, whereas the hydroxybenzoic acid derivatives are present in their free forms [1]. The North American Native grape *Muscadinia rotundifolia* (Michx.) Small, “Ison” var., was reported to have a higher gallic acid content (between 7 and 10 fold higher) than the European grape (*Vitis vinifera* L., “Chardonnay” and “Merlot” var., respectively) [28]. Gallic acid has been shown to possess various therapeutic properties, including antioxidant, anti-cancer, anti-inflammatory, antifungal and antiviral activities [29,30,31,32]. However, polyphenols including flavonoids, stilbenes and proanthocyanidins are the most important class of biologically active compounds in grapes. Grape is one of the richest sources of polyphenols among fruits. The flavonoids are the most abundant biologically active phytonutrients among the polyphenols found in grapes, possessing cardioprotective, neuroprotective, antimicrobial and antiaging properties [26,33,34,35,36]. Most of the flavonoids are found primarily in the outer epidermal cells (the grape skin), whereas about 60%–70% of total polyphenols are stored in grape seeds [1,25,37]. During processing of grape juice, only limited amounts of anthocyanins (~2%) are extracted with the cell sap [38]. However, when fermentation/maceration processes are involved, large amounts of polymeric products are obtained including proanthocyanidins, pyranoanthocyanins (vitisin A and vitisin B) and oligostilbenes (ε-viniferins and δ-viniferins). These polymeric compounds also increase the color stability and biological values of the resulting wines [39]. Nevertheless, more than 70% of grape polyphenols remain in the pomace (a byproduct of wine/grape juice processing), which becomes a valuable source of health promoting nutraceuticals [40]. Moreover, grape seeds may be separated from the pomace and used either for production of grape seed oil or as individual food supplements in the form of grape seed powder or grape seed extracts [41]. Additional flavonoids-rich products are also extracted from grape skins [42,43]. The North American grape species, such as *M. rotundifolia* (Michx.) Small and *V. labrusca* L. accumulate ellagic acid in their berries [44,45]. Recently, ellagic acid has attracted the increased attention because of its high antioxidant, anticarcinogenic, antimutagenic and hepatoprotective qualities [46]. The presence of ellagic acid significantly increase the nutraceutical value of food additives produced by the native North American grapes, compared to the similar products obtained by the processing of regular European grape species. Primary products and byproducts from the processing of fresh grapes used as supplements or in regular diets may provide significant health benefits for humans (Figure 1). Therefore, the wide range of pharmacological effects of grapes and grape products on human health is due to the fact that those additives are sources of unique combinations of nutraceuticals.

### 2.1. Flavonoids in Grapes

**Figure 2:**
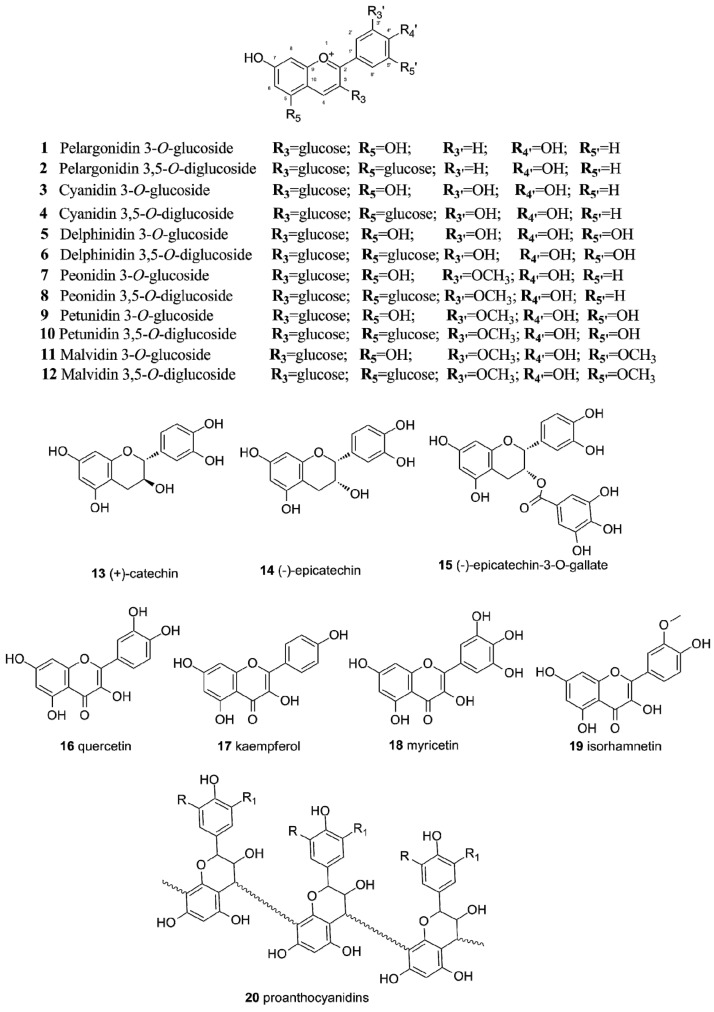
Structures of some common grape flavonoids.

Flavonoids represent a large family of secondary metabolites and nearly 6000 structures have been identified in plants [47]. The diversity in their chemical structures contributes to their broad range of physiological and biological activities. The most common flavonoids found in grapes are anthocyanins (3-*O*-monoglucosides or 3,5-*O*-diglucosides of malvidin, cyanidin, peonidin, delphinidin, pelargonidin and petunidin **1**–**12**, as well as their acetyl-, p-coumaroyl- and/or caffeoyl-esters), flavonols (3-*O*-glycosides of quercetin **16**, kaempferol **17**, myricetin **18**, laricitrin, isorhamnetin **19** and syringetin), flavanols [(+)-catechin **13**, (−)-epicatechin **14**, (−)-epicatechin-3-*O*-gallate], dihydroflavonols (astilbin and engeletin) and proanthocyanidins **20** (Figure 2) [1,3,25,26,27,48]. Anthocyanins are found only in red grape varieties. They accumulate mainly in the berry skin, but in some varieties known as “teinturier” (or dyed), anthocyanin pigments are found to accumulate in the flesh of the berry [48,49]. It is important to note that there is a close correlation between anthocyanin biosynthesis and berry development: it starts at “veraison” when proanthocyanidin biosynthesis is concluded and reaches maximum level at berry “maturity” [50]. Each grape species and variety respectively has a unique set of anthocyanins [51]. European grapes produced only anthocyanidins 3-*O*-monoglucosides, whereas muscadine grapes produced only anthocyanidins 3,5-*O*-diglucosides [51].

Flavanols are present in grapes mainly in the form of (+)-catechin **13**, (−)-epicatechin **14**, and proanthocyanidins **20**. They accumulate in the grape seeds, but are also found in the skin of the grape berries [52]. In white grape varieties flavanols represent 46% to 56% of total phenolics, whereas in red grapes they represent between 13% and 30% of total phenolic content [52]. As the second most abundant flavonoids in grapes, flavonols are present only as 3-*O*-glycosides in grape skins, but can be found also as aglycones (quercetin **16**, kaempferol **17**, myricetin **18**, isorhamnetin **19**) in wines and juices as a result of acid hydrolysis during processing and storage [53]. Quercetin, kaempferol and isorhamnetin derivatives are found in both red and white grapes, whereas myricetin derivatives are found only in red varieties [51,53]. The profile of flavonols strongly depends on grape cultivars, but in general quercetin-3-*O*-glucoside and quercetin-3-*O*-glucuronide are the predominant compounds present in most grapes [51]. In muscadine grapes, quercetin-3-*O*-rhamnoside and quercetin aglycone have been identified as the major flavonols [51]. The biological activities of flavonoids have been determined to strongly depend on several factors such as the degree of glycosylation, type of sugar residues and subsequent acyl esterification [54]. It is therefore possible to select different grape cultivars with unique flavonoid patterns having different health promoting effects on the human body.

### 2.2. Antioxidant Action

**Figure 3:**
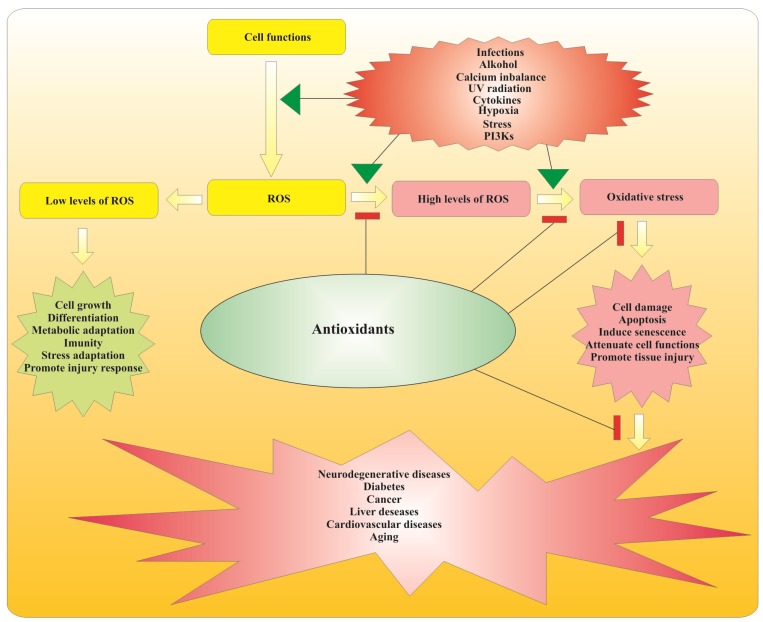
Reactive oxygen species (ROS) and their contribution to development of oxidative stress-related diseases. The presence of antioxidants may prevent diseases development by modulating damaging effects of ROS by suppressing multiple process steps.

As a result of their aerobic metabolism, active cells produce toxic byproducts known as reactive oxygen species (ROS). ROS account for a wide range of aggressive free radicals produced by various metabolite pathways in living cells [55]. Under normal physiological conditions, ROS are considered to be important regulatory agents in the complex signaling network of cells. They play a major role in promoting cell growth and differentiation, adaptation to metabolic and physiological stresses, immune response as well as protection from pathogen invasion [55,56,57,58]. However, several factors may cause an over-accumulation of ROS by interrupting regular cellular processes and thus exposing tissues to conditions of oxidative stress (Figure 3).

When cells are exposed to oxidative stress they easily undergo oxidative damage that leads to a cascade of degenerative processes. Development of severe pathologies including neurodegenerative diseases, diabetes, cancer, liver diseases, cardiovascular diseases, and rapid aging are among the possible outcomes [57,58,59,60]. It is generally assumed that therapeutic treatment with antioxidants is the most effective way to control oxidative stress and to avoid occurrence of oxidative damage [57,60,61]. Various molecules by weight and structure including enzymes and hormones may have antioxidant activities in biological systems [62]. Antioxidants may neutralize oxidative stress in various ways including inhibition of free radical formation (preventive antioxidants), interrupting autoxidation chain reactions (chain breaking antioxidants), up-regulating and protecting cellular antioxidant defenses mechanisms (indirect antioxidants), neutralizing the action of metal pro-oxidant ions (metal chelators), inhibiting the action of pro-oxidative enzymes (enzyme inhibitors) and increasing the activities of other antioxidants (synergistic compounds) [63,64].

Flavonoids represent a large group of low molecular weight compounds with high antioxidant properties. Their specific chemical structure allows them to reduce oxidative stress through numerous mechanisms [34,65]. For example, it was reported that, *in vitro*, flavonoids could act both as preventive antioxidants and chain breaking antioxidants [scavenging superoxide, peroxyl, alkoxyl and hydroxyl radicals as well as preventing low-density lipoprotein (LDL) oxidation] [66]. On the other hand, flavonoids can also act as metal chelators (reducing ferric and cupric ions), and enzyme inhibitors (inhibiting the enzymes involved in ROS generation: xanthine oxidase; protein kinase C; cyclooxygenase; lipoxygenase; glutathione S-transferase; microsomal monooxygenase; mitochondrial succinoxidase and NADH oxidase) [66]. However, *in vivo* research has also demonstrated that flavonoids also may act as indirect antioxidants by up-regulating antioxidant defense system and increasing uric acid concentration in the plasma [63,64,66]. Therefore, as a result of their metabolic conversion in the human body, flavonoids generate large amounts of simple phenolic acids, which have significant effects in scavenging free radicals and improving the action of other antioxidants [66]. The consumption of grape-derived dietary flavonoids in the form of grape extracts and grape seed powders has been shown to effectively suppress oxidative stress and prevent oxidative damage *in vivo* [67,68,69]. Such activities are attributed to various functions of grape flavonoids as free radical scavengers and metal chelating compounds [67,68,69].

### 2.3. Anti-Inflammatory Action

**Figure 4:**
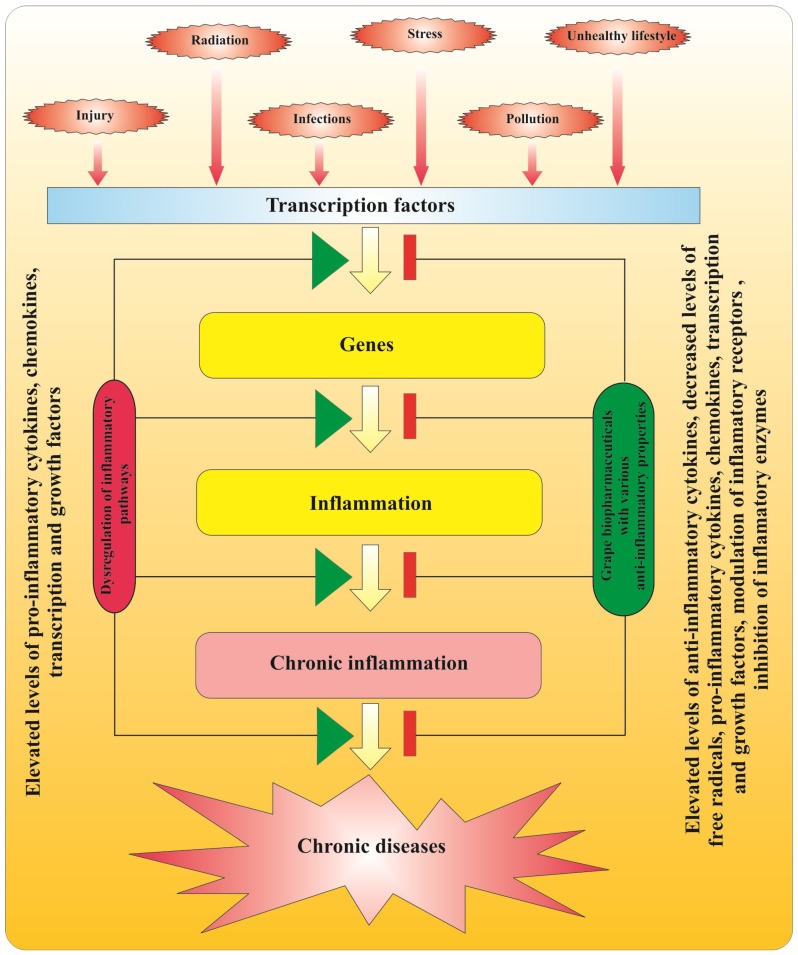
Principal way of development and modulation of chronic inflammation-related diseases.

Inflammation is a protective response of tissues against cell injury, irritation, pathogen invasions, as well as mechanism for eliminating damaged and necrotic cells [70,71]. Several environmental stress factors may cause inflammation (Figure 4). Under normal physiological conditions, a short period of acute inflammation can overcome negative effects on injured tissue. However, if inflammation is prolonged, chronic inflammation can developed [70,72]. Chronic inflammation is considered to be the main mediator in the development of chronic diseases such as cancer, Alzheimer’s, neurodegenerative diseases, cardiovascular diseases, diabetes, arthritis, autoimmune and pulmonary diseases [70,71,72,73,74,75]. Basic steps in progression of chronic inflammation are presented on Figure 4. When signal-dependent transcription factors [nuclear factor κB (NF-κB) and activating protein 1 (AP-1)] are activated, they induce the expression of genes involved in inflammatory response [75]. Intensive production and secretion of pro-inflammatory cytokines and chemokines, once started, can form concentration gradients in affected tissues, which may lead to the amplification of the initial inflammatory response [70,72,75,76]. As a result, additional immune cells are recruited and increased levels of ROS are produced [70,75,76,77]. Under normal physiological conditions, anti-inflammatory cytokines act as immunoregulators to control the inflammatory reactions [70,77]. Deregulation of precise control mechanism of inflammation leads to chronic inflammation and promotion of chronic disease (Figure 4). Grape polyphenols have been shown to decrease chronic inflammation either by modulation of inflammatory pathways or by reducing ROS levels. As natural compounds, grape flavonoids and proanthocyanidins can target multiple pathways to overcome chronic inflammation, and thus are more effective compared to synthetic mono-targeted anti-inflammatory drugs [78]. Freeze-dried extract of wine from “Jacquez” grapes (*Vitis aestivalis-cinerea* × *Vitis vinifera*), which contains mainly flavonoids, anthocyanins, proanthocyanidins and hydroxycinnamic acid derivatives, showed higher anti-inflammatory activity when compared to the commercial non-steroidal anti-inflammatory drug (NSAID) indomethacin [79]. It has also been demonstrated that proanthocyanidins in grape seeds have high anti-inflammatory action, because they scavenge free radicals, prevent lipid peroxidation and inhibit formation of pro-inflammatory cytokines [80].

Proanthocyanidins extracted from the grape seeds have also been found to have an immune-modulatory role in inflammatory conditions that exert an overproduction of nitric oxide and prostaglandin E2 [81]. The suppression effect of extracts obtained by red and white grape pomaces on chronic inflammation induced by lipopolysaccharide and galactosamine, has been investigated *in vivo* [82]. The authors found that the extract of red grape pomace suppresses the activation of inflammatory transcription factor NF-κB and thus could be used as raw material for both extraction of new anti-inflammatory candidates or as an additive in processing functional foods [82].

### 2.4. Antimicrobial and Antiviral Activities

Antimicrobial activities of grape, wine and grape-derived byproducts have been widely discussed [26,83]. It has been demonstrated that grape seed extract of *V. vinifera* “Bangalore Blue Grapes” var. exhibits stronger antibacterial activity against Gram-positive bacteria compared to the response from Gram-negative bacteria [84]. Grape juice, skin and seed extracts from black table grapes “Ribier” var. have also been found to have a strong inhibitory effect against the growth of *Listeria monocytogenes* [85]. In addition, research has also shown that red colored (anthocyanin pigments) grape juice and skin extract have pH-dependent anti-listerial activity, while the seed extract showed pH-independent anti-listerial activity [85]. Strong anti-listerial activities have also been demonstrated for extracts of grape berries, seeds, pomace, and stems from red (*V. vinifera* “Mandilaria” and “Voidomato” var.) and white (*V. vinifera* “Asyrtiko” and “Aidani” var.) grape varieties. The main active compounds were catechine, epicatechine and epicatechine galate [86]. Recent research reported effective antiviral activity of grape skin extracts from fermented “Pinot Noir” var. and non-fermented “Pinot Gris” var. pomace against influenza virus [87]. Phytochemical analyses of the grape varieties from the study showed that the major phenolics in “Pinot Noir” var. extract were catechin and epicatechin, whereas rutin, catechin and epicatechin were predominant in extract of “Pinot Gris” var. [87]. However, no significant difference was observed between antiviral activities of both extracts, which suggested that they may be equally used as additives in different functional health promoting beverages [87]. Alcohol-free red and white wine extracts have been shown to have moderate antifungal activities on *Candida albicans* depending on their total phenolic contents [88]. Extracts from pomace, fermented seeds and skin of “Pinot Noir” var. were found to be more effective against *Candida albicans* than the ones from the “Pinot Meunier” var. [89]. Therefore, the observed antifungal activity of grape products made them attractive for commercial application, and some are being incorporated into skin care cosmetics. In addition to observed antimicrobial properties, grape flavonoids may play an important role in modulation of human gut microflora. Research has demonstrated that red wine grape extract (two parts of red wine extract and one part of grape juice extract) significantly reduced the ratio of Firmicutes: Bacteroidetes in an *in vitro* model simulator of the intestinal microbial ecosystem and thus could have beneficial effects in control of weight loss [90].

## 3. Bioavailability and Absorption of Flavonoids

Studies investigating the bioavailability of anthocyanins that looked into the pharmacokinetics, absorption, metabolic fate and excretion have increased exponentially over the past decade in both human and animal models [91]. In the process of studying the bioavailability of anthocyanins, animals or volunteers are generally administered with anthocyanin-rich foods. For instance berries, juices, wines, extracts or pulps can be administered. After a certain period, plasma and urine are collected for analysis. In most studies focusing on anthocyanins, the resulting peak concentrations of pigments in plasma vary from the mid to extremely low levels [92]. Garcia-Alonso *et al.*, [93] reported an even lower amount of anthocyanins (0.05% of ingested dose), found in urine after the consumption of 12 g grape peel extract (equivalent to 183.9 mg total anthocyanins). However, even with low absorption capacity, grape polyphenols can have direct positive impact on gut mucosa [94]. Feeding experiments based on oral administration of wine to rats showed that poorly adsorbed grape polyphenols may decrease the oxidative damage exerted on DNA in caecal mucosal cells [94]. These studies suggest that some dietary flavonoids can exert a positive effect regardless of their poor absorption. However, it is important to realize that the most common flavonoids in the human diets are not necessarily the most active within the body, either because they have a lower intrinsic activity or because they are poorly absorbed from the intestine, highly metabolized, or rapidly eliminated [95]. In addition, the flavonoids that are found in blood and target organs and that result from digestive or hepatic activity may differ from the native substances in terms of biological activity [95]. Extensive knowledge of the bioavailability of flavonoids is thus essential if their health effects are to be understood. However, the beneficial activities of dietary flavonoids as antioxidants and/or anti-inflammatory agents could be under question in the human body, since these abilities are dependent on their structural properties, and currently very little is known about the bioavailability and further metabolism of those important nutraceuticals. Flavonoids are thought to be poorly absorbed because they occur in nature in highly hydrophilic glycosylated forms. However, only aglycones can effectively pass through the gut wall. It has been suggested that some species of colon microflora can hydrolyze flavonoid glycosides to their corresponding aglycones, although the microbial enzymes may also degrade the entire compound as well [96].

## 4. Factors Affecting Bioavailability of Flavonoids

The diverse results in the area of bioavailability, including plasma and urine concentrations, as well as the availability of certain compounds can be attributed to factors affecting bioavailability of flavonoids. Some of the most important factors to consider are sources of flavonoids, specific chemical features of flavonoids, type of the food matrix, dosage, individual variation, analytical methodology, and detection minimum [92,97]. A recent study by Charron *et al.* [97], investigated the bioavalability of purple carrot juice anthocyanins, depending on the dosage, molecule acylation, and plant matrix. In their experiment, the highest concentration of anthocyanins in plasma was observed after consumption of 250 mL (323 mg total anthocyanins) carrot juice and it was found to decline in a dose-dependent manner, when lower doses (50 and 150 mL) were used. The authors found that the bioavailability of nonacylated anthocyanins was significantly higher, when compared to that of acylated anthocyanins. Moreover, the increased administration dose resulted in decrease in absorption efficiency and the authors suggested that saturation equilibrium of cyanidin-based anthocyanins was between 250 and 350 µM [97]. Therefore, increasing the dosage of consumed flavonoid compounds can result in improved plasma concentration but only until a saturation point is reached [98]. In addition to the above mentioned factors, sample preparation techniques and modifications of molecule structure can also contribute to the incensement on overall recovery of flavonoids as demonstrated by Woodward *et al.* [99].

## 5. Grape Flavonoids and Human Health

Currently, there are thousands of grape-derived products on the market including juices, wines, jam, jelly, raisins, and others. Recently, it was found that even byproducts, obtained as a result of grape processing (pomace, seeds, skins, seed oil) have high nutraceutical values and were commercialized in various forms of different powders, granulates, concentrated or dried extracts and other innovative means of packaging. Here some recent scientific facts, concerning the effects of these products on body health status are briefly discussed.

### 5.1. Brain Function

Consumption of flavonoid-rich grape products may have a significant beneficial effect on brain function and central nervous system [100]. Grape flavonoids, specifically anthocyanins, can prevent neurodegenerative processes both by inhibition of neuro-inflammation and by reducing oxidative stress. A clinical study demonstrated that 12 weeks supplementation with *Vitis labrusca* “Concord” var. grape juice in the diet may have neurocognitive benefits in older adults with early memory decline [101]. Consumption of “Concord” var. grape juice was also found to improve memory functions in older adults with mild memory decline [102]. Recently, it was demonstrated that polyphenol-rich grape seed extract has a significant capability of disrupting and disintegrating the ultrastructure of native paired helical filaments (a key neuropathological feature in Alzheimer’s disease) [103]. The authors showed that resveratrol was ineffective in this process but rather catechin and epicatechin were involved [103].

### 5.2. Obesity and Diabetes

Metabolic syndrome–related diseases and obesity are the most prevalent nutrition-related issues in the United States [104]. Evidence suggests that polyphenols in grapes and grape products may reduce metabolic syndrome and prevent development of obesity and type 2 diabetes, by acting as multi-target modulators with antioxidant and anti-inflammatory effects [100,104]. Freeze-dried grape powder and grape powder extracts, obtained from red, green, and blue-purple seeded and seedless California grapes were tested for their effects on glucose tolerance and inflammation in obese mice [105]. The authors found that grape powder acutely improves glucose tolerance and chronically reduces inflammatory markers in obese mice [105]. They also reported that quercetin-3-*O*-glucoside was the compound with the highest bioavailability in grape powder extracts and can reduce several inflammatory markers in human adipocytes [105]. Animal model study showed that, in addition to the currently known anti-inflammatory and antioxidant activities, grape seed extract prevents metabolic syndrome, type 2 diabetes and obesity, also by modulating of metabolic endotoxemia and improving of gut barrier integrity [106].

### 5.3. Herpatoprotective Activity

Environmental factors such as pollutants, alcohol, viral infections, and aflatoxins, can promote development of liver disease [60]. Grape polyphenols have the ability to protect liver because of their anti-inflammatory and antioxidant properties [25]. Polyphenol-rich grape skin extract has been found to improve liver steatosis and to protect against diet-induced adiposity and hepatic steatosis [107]. These effects were probably due to the suppression of lipogenic enzymes in liver and adipose tissues and modulation of lipid metabolism by regulation of mRNA expression of enzymes, involved in regulation of lipogenesis and fatty acids oxidation [107]. Another study compared the protective effects of aqueous and ethanol seed extracts of red grapes against ethanol-induced cytotoxicity in the liver [60]. The research revealed that ethanol grape seed extracts was more effective against hepatotoxicity of alcohol, when compared to aqueous grape seeds extract. The authors attributed the observed effect to the nature and antioxidant activity of extract’s constituents [60].

### 5.4. Cardiovascular Diseases

Several studies have shown that consumption of grape products may have beneficial effect on cardiovascular system by enhancing endothelial function, decreasing LDL oxidation, improving vascular function, altering blood lipids, and modulating inflammatory process [23,24,25,26,100]. It has also been demonstrated that consumption of flavonoid-rich purple grape juice may attenuate cardiovascular diseases and inhibit thrombosis [108]. Clinical study suggested that this effect is probably due to the suppression of platelet-dependent inflammation by significant decrease in levels of platelet-dependent superoxide and the inflammatory mediator sCD40L after consumption of purple grape juice [108]. Recently it was demonstrated that consumption of resveratrol-rich grape extract could exert additional vascular protective benefits in stable patients with coronary artery disease, when compared to the action of a conventional grape extract or a placebo [109]. A one year clinical study demonstrated that regular consumption of resveratrol-rich grape extract increased serum adiponectin, prevented incensement of plasminogen activator inhibitor type 1 (PAI-1) and inhibited atherothrombotic signals in peripheral blood mononuclear cells [109]. Recent research showed that consumption of grapes has anti-oxidative effect and increases the levels of anti-inflammatory factors in the absence of dyslipidemias in men with metabolic syndrome [110].

### 5.5. Cancer Prevention

Anticancer properties of grapes and grape products have been widely discussed in the scientific literature [1,25,26,111]. The remarkable anticancer effect of grape products is considered to be due to their unique mixture of polyphenolic compounds with various biological activities [111]. Flavonoids are the main group of active anticancer constituents in grape products, and are concentrated mainly in grape skins and seeds [111]. Researchers have shown that grape skin extract possesses chemotherapeutic efficacy against breast cancer with metastases in model system [112]. Recently, extracts of raisins from two grape varieties (*V. vinifera* “Currant” and “Sultana” var.) were investigated for their effect on human colon cancer cells [113]. The authors found that both extracts exhibited cancer preventive efficacy on colon cancer cells by having antioxidant and anti-inflammatory effects [113]. Treatment of human pancreatic cancer cells with grape seed proanthocyanidins significantly reduced cell viability and induced apoptosis in a dose- and time-dependent manner [114]. The authors demonstrated that grape seed proanthocyanidins can inhibit migration of human pancreatic cancer cell by inactivating the inflammatory transcription factor NF-κB [114].

## 6. Plant Cell Suspensions as Alternative Source of Grape Flavonoids

**Figure 5:**
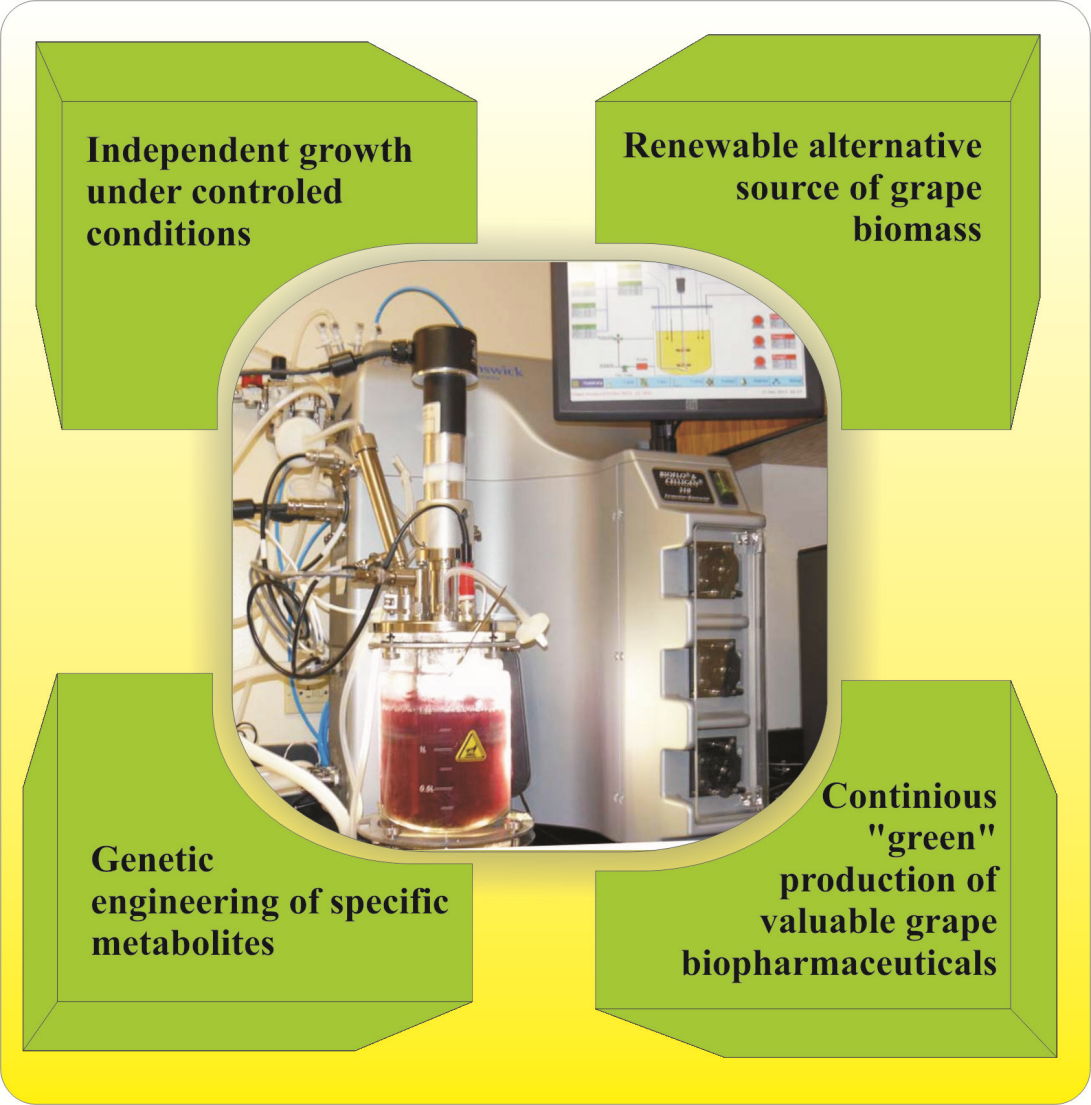
Advantages of *in vitro* grape cell suspension cultures as a source of nutraceuticals.

Plant cell cultures are generally considered as prospective alternatives for continuous production of phytochemicals under controlled conditions [115,116,117]. In the last few years, plant cell biotechnology of grapes and especially grape cell suspensions has enjoyed great attention from both science and industry [27]. Grape cell suspensions have been used to study the mechanism of secondary metabolite biosynthesis, for conducting functional genetic studies, somatic embryo development and *in vitro* production of valuable polyphenols [27,118,119,120,121,122]. Growth of grape cells in bioreactors for production of biologically active nutraceuticals exerts several advantages compared to conventional breeding (Figure 5). Strictly controlled environmental conditions during *in vitro* cultivation of grape cells minimize variation in yield and unpredictable metabolite composition that occurs with natural harvest. Production of nutraceutical-rich grape biomass can be done continuously throughout the year without concern about the seasons, soils and climate changes. Several optimization strategies may be involved for increasing the yields of the targeted phytochemicals, thus concentrations may exceed levels in the normal plant. For example, a non-resveratrol producing grapevine cell suspension of grape hybrid (*Vitis vinifera* “Chasselas” × *Vitis berlandieri*) elicited with 0.2 mM methyl jasmonate was found to initiate resveratrol production [123]. When the culture was scaled-up to a laboratory bioreactor, a total resveratrol yield of 230 mg/L was achieved. In addition, 90% of produced resveratrol (209 mg/L) was found to be secreted into the culture medium [123].

The ability of grape cells to secrete and accumulate secondary metabolites in culture medium is of high technological importance because this may significantly facilitate the downstream processing of target phytochemicals [116]. The development of reliable and sustainable sources of highly purified resveratrol is of special interest for the pharmaceutical industry because this compound may exert both estrogenic and anti-estrogenic effects *in vitro* and *in vivo* [124]. A recent study demonstrated that treatments with high concentrations of resveratrol may have cancer chemopreventive and therapeutic effects, the intake of resveratrol in low concentrations as a dietary supplement may also have promoting effect on mammary tumor growth and metastasis [125]. Thus, to achieve therapeutic effect and to eliminate the risk of side effects, resveratrol should be administrated only in high concentrations. Resveratrol-rich grape biomass, produced by *in vitro* cultivation of grape cells could be used as a potential natural additive to overcome this complication. In addition to the increased dose of resveratrol, the grape cells biomass can also provide the full unique mix of all naturally occurring nutrients found in field-growing grape, which usually have emphatic synergistic effect on its biological activity.

Flavonoid biosynthesis in grape cell suspensions is extremely sensitive to composition of nutrient medium. Studies using plant cell suspension of *V. vinifera* L. “Gamay Fréaux” var. Tenturier showed that two types of media (maintenance and production) should be used to support the best growth rate and to achieve the highest flavonoid production [126]. When cultivated on maintenance medium (low sucrose and high nitrate contents) the cells have maximum growth rate but extremely low levels of flavonoid accumulation [126]. When sucrose, ammonium, phosphate and magnesium concentrations in nutrient medium were increased (3, 2, 2 and 2 folds, respectively) the maximum yields of anthocyanins (1100 mg/L), proanthocyanidins (300 mg/L) and catechins (25 mg/L) were achieved [126]. Catechins [(+)-catechin, (−)-epicatechin and epicatechin 3-*O*-gallate], isolated from *V. vinifera* L. “Gamay Fréaux” var. cell suspensions were found to have cancer-chemopreventive effects by inhibiting cyclooxygenases (COX-1 and COX-2) activities, whereas the stilbenoids isolated from the same culture were more potent in inhibiting the development of 7,12-dimethylbenz[*a*]anthracene (DMBA)-induced preneoplastic lesions [127]. It is possible that common media manipulations may have significant effects on phytochemical patterns of *in vitro* growing grape cells. Adaptation of the well-established plant cell techniques may contribute to development of sustainable technology for production of improved grape cell biomass. It could be expected that such biomass will contribute to development of natural products with increased added value, since it contains the same unique mix of biologically active compounds compared to those found in regular growing grapes but in higher concentrations. However, the technology is still under investigation and more research and clinical studies are needed to strengthen the nutritional and beneficial health effects of *in vitro* produced grape cell biomass.

Another potential application of grape cell suspension is in production of marker compounds, used in *in vivo* study of bioavailability and metabolism of phytonutrients. Cell suspension culture of *Vitis* hybrid “Bailey Alicant A” [(*V. lincocumii* × *V. labrusca* × *V. vinifera*) × (*V. vinifera* × *V. vinifera*)] was used to produce radiolabeled anthocyanins, (+)-catechin and (−)-epicatechin by feeding with ^14^C labeled sucrose during cultivation [128]. The production of radiolabeled flavonoids by grape cell suspension have great potential as a reliable source of labeled phytochemicals used for *in vivo* study of bioavailability, catabolism and accumulation of these chemicals in animals and humans. Further improvement of production yield of labeled flavonoids could significantly decrease the price and increase the availability of such rare and expensive isotopic markers.

Despite all the positive effects of plant cell technology, the cultivation of grape cells is still carried out in small scale within the laboratory. The slowdown in scale-up has mainly been caused by the relatively low yields and expensive equipment, which decrease the economic effectiveness of the entire process. Genetic engineering may be applied to achieve additional increase of grape cell culture productivity. Genetic manipulation of the flavonoid biosynthetic pathway is a powerful tool for increasing the yields of targeted products or redirecting cell metabolism to produce only the desired group of metabolites [3,27]. Moreover, *in vitro* cultivation of grape cells in an eco-friendly bio-safe environment is of great importance. Hence, the process ensures the complete isolation of genetically modified cells from the natural environment and eliminates the risk of cross-contaminations and transgene migration [117].

## 7. Nutraceutical Products Derived by Grapes

Wine is the most popular and widely discussed nutritional grape product with proven beneficial health effects on human body. Moderate consumption of red wines in daily diet (often referred as “Mediterranean way of drinking”) is considered to contribute for overall improvement of consumers’ health, mainly due to the beneficial effects of quercetin and resveratrol [129]. However, wine also contains alcohol, which significantly restricts its mass consumption especially among underage and actively working individuals. Studies have shown that the consumption of red wine can increase antioxidant activity of plasma. However, over-consumption may lead to the increase of isoprostanes—an oxidative lipid damage marker [130]. The authors demonstrated that this effect may be avoided by consumption of dealcoholized red wine, which has the same flavonoid compounds (myricitin, quercetin and isorhamnetin) and in similar concentrations as in red wine, but is free of alcohol [130]. The unique combination of grape polyphenols, including flavonoids, anthocyanins, proanthocyanins, and stilbenes, makes grape a promising source for the development of novel nutraceutical products. In the last few years, there has been a wide range of food additives and nutritional products originating from grapes, distributed in the worldwide market. Most of these commercialized products are obtained during processing of pomace from wine or grape juice production. This includes several grape skin or seed extracts, grape skin powder, dry seed powder (capsulated or bulk), pomace powder, and anthocyanin colorants. The diversity of these products, and their biological activities and health benefits are reviewed by Amarowicz and Weidner [131]. Recently, the biomass from grape cell suspension of *V. vinifera* L. “Gamay Fréaux” var. was commercialized by the Swiss company “Mibelle Biochemistry”. The purpose of the commercialization was to develop a natural additive “PhytoCellTec™ Solar Vitis” for exclusive application in skin-care and other cosmetic products [132]. This is the first commercial grape product in the market, obtained by the methods of modern high-end plant biotechnology. According to the company, the product is rich in anthocyanins and its application may have beneficial effects on skin because of the presence of strong UV protectors and anti-aging ingredients.

## 8. Conclusions

Grape and grape products should be promoted in our daily diet not only as a nutrient, but as a healthy food as well. Currently, a growing number of researchers are focusing on the biological activities of grape and grape products as prospective sources of valuable nutraceuticals. Numerous studies have strongly suggested that the inclusion of grapes and grape products as supplements in our daily intake of foods may generate significant health benefits. However, to achieve beneficial therapeutic effects, most of these phytochemicals must be used in a strict dose-dependent manner. Bioavailability in *in vivo* conditions is a key issue to be resolved for establishing the level of therapeutic blood concentrations of grape flavonoids. Therefore, more studies are needed in this area. Future development of new renewable sources, such as *in vitro* cell systems that can continuously produce highly purified grape flavonoids is essential and can be broadly applicable.
